# Psychological distress among elderly surgical patients who had their surgery postponed during the COVID-19 pandemic

**DOI:** 10.1186/s13741-022-00242-7

**Published:** 2022-03-17

**Authors:** Pui San Loh, Sook Hui Chaw, Yi Xian Foong, Dhurgka Ramasamy, Rafdzah Ahmad Zaki, Shanggar Kuppusamy, Teng Aik Ong, Mee Hoong See, Hui Min Khor

**Affiliations:** 1 Department of Anaesthesiology, Universiti Malaya, 50603 Kuala Lumpur, Malaysia; 2 Department of Social and Preventive Medicine, Universiti Malaya, 50603 Kuala Lumpur, Malaysia; 3 Department of Surgery, Universiti Malaya, 50603 Kuala Lumpur, Malaysia; 4 Department of Medicine, Universiti Malaya, 50603 Kuala Lumpur, Malaysia

**Keywords:** Psychological distress, Anxiety, Depression, Elective surgical procedures, Elderly, COVID-19

## Abstract

**Background:**

Many institutions withheld elective lists and triaged surgeries during the peak of coronavirus disease 2019 (COVID-19) pandemic. As a result, older surgical patients have had to wait for rescheduled dates in a long waitlist. This study aimed to identify the psychological impact in these patients when they returned for surgery.

**Methods:**

This was a cross-sectional study which included 153 patients aged ≥ 65 years undergoing elective surgery. Trained interviewers recruited and assessed psychological status pre-operatively with two validated questionnaires - Hospital Anxiety and Depression Scale (HADS) and 36-item Short Form Health Survey (SF-36). Specific questions were asked about their postponed surgeries, appetite and fear.

**Results:**

A total of 36 out of 153 (23.5%) patients had their procedures deferred during the first wave of COVID-19 pandemic. Postponed cases were significantly based on the nature of surgery (*p* = 0.002), cancer diagnosis (*p* = 0.006) and surgical specialty (*p* = 0.023). Median HADS scores were higher for patients who were postponed (2.00 versus 4.00 for anxiety, *p* = 0.180 and 0.00 versus 1.00 for depression, *p* = 0.424) although no statistical significance was shown. In the whole study population, anxiety was a significant predictor for depression and vice versa (*p* < 0.001) with other predictive risk factors for anxiety that were age ≥ 85 years old (odds ratio [OR] 6.14, *p* = 0.018), female (OR 2.41, *p* = 0.024), cancer (OR 2.19, *p* = 0.039) and major surgery (OR 2.39, *p* = 0.023). Similarly, older patients ≥ 85 years old (OR 10.44, *p* = 0.003) and female (OR 6.07, *p* = 0.006) had higher risk for depression. Both anxiety and depression were significant risks for loss of appetite (*p* = 0.005 and 0.001). Lastly, the fear of disease progression due to rescheduling was more frequent in cancer patients (*p* = 0.035).

**Conclusion:**

The mental health and disease burden of older surgical patients should be taken into careful consideration when cases need to be postponed in our healthcare system.

**Supplementary Information:**

The online version contains supplementary material available at 10.1186/s13741-022-00242-7.

## Background

At the height of the coronavirus disease 2019 (COVID-19) pandemic during the first wave, many routine hospital services were suspended to conserve resources and manpower worldwide. A global expert-response study estimated that over 28 million operations were cancelled or postponed during the peak 12 weeks of disruption with overall cancellation rate of 72.3% (COVIDSurg Collaborative, 2020). Cases that were most affected were those benign in origin (81.7%) and cancer-related surgeries (37.7%). By late March 2020, recommendations were made to raise thresholds for elective surgery in a joint news release by University of Birmingham and College of Surgeons with the Academy of Medicine of Malaysia with validated policy decisions at national and institutional levels to postpone non-urgent surgeries (COVIDSurg Collaborative, 2020; Nepogodiev et al., 2020; University of Birmingham, College of Surgeons, Academy of Medicine of Malaysia, 2020). To put perspective into the consequence of that decision in many regions, a median of 45 weeks was the predicted duration needed to clear the backlog of cases if affected countries increased their normal surgical volume by 20% in the post-pandemic period (COVIDSurg Collaborative, 2020).

As approximately 53% of all surgical procedures are performed on patients over the age of 65, the elderly group may be the most affected surgical subpopulation (Yang et al., 2011). The delay in surgery for them, regardless of the clinical diagnosis, would be a dilemma as the risk of delaying surgical treatment and admission had to be weighed against the risks of getting admitted for surgery and acquiring hospital-transmitted severe acute respiratory syndrome coronavirus 2 (SARS-CoV-2) infection that led to severe acute respiratory syndrome (COVIDSurg Collaborative, 2020; Richterman et al., 2020). Moreover, when the patterns of surgical care were analysed, the most common elective procedures were reported as lens and cataract procedures, joint replacement surgeries, urology and colorectal surgeries (Deiner et al., 2014). The former two were most likely postponed while the others  would have been given priority and continued if they were cancer-related.

In addition, the pandemic itself presented another unique challenge to older patients. The need to practice social isolation in order to stem the spread of the virus while maintaining social connections to ensure psychological well-being was nevertheless a difficult accomplishment (Gossage, 2020). In older adults, social isolation predicted mortality and other adverse outcomes (Jaul & Barron, 2017; Steptoe et al., 2013). The diagnosis of cancer, a common indication for surgery and the leading cause of death from age 40 to 79 years old, also added to the burden in elderly patients (Jemal et al., 2010).

Although the adverse effects of quarantine and social isolation on individuals’ well-being are well documented (Brooks et al., 2020), little is known about older patients who require surgery but have had their surgery postponed because of the COVID-19 pandemic. Moreover, the extent of psychological impact created by the change in their management has not been examined. Therefore, this study aimed to study the extent of postponed elective surgeries during the first wave of pandemic under full COVID-19 lockdown in 2020 among geriatric patients who returned for their surgeries 3 months later and to determine the physical and psychological impact of this medical decision. Our secondary aim was to identify the same impact on postponed cancer surgeries.

## Methods

### Ethics

The study was approved by the Medical Research Ethics Committee of University of Malaya Medical Centre (Ethics approval number: 2020623-8801) and registered in ClinicalTrial.gov (ClinicalTrials.gov identifier: NCT04547218). All patients gave written informed consent before the study.

### Study design

This was a cross-sectional study conducted in 2020 when elective surgical lists were restarted fully after 12 weeks of restricted operating services during full COVID-19 lockdown. Analysis and reporting of this study followed the STROBE (Strengthening the Reporting of Observational Studies in Epidemiology) guidelines (von Elm et al., 2007).

### Setting

The study was performed in a university-based tertiary centre with 1600 bed occupancy and 35 operating theatres in total.

### Participants

Geriatric patients aged ≥ 65 years who were planned for elective surgeries under general anaesthesia (GA), regional anaesthesia (RA) and monitored anaesthetic care (MAC) were screened for eligibility and recruited. Those who refused consent or had cognitive dysfunction evidenced by Mini-Mental State Examination (MMSE) score < 24 were excluded. This study also did not include any emergency or trauma cases.

As there has been no existing literature, the sample size depended on time-based recruitment via convenience sampling. An estimate from an audit on the local surgical population indicated 28% of electively listed patients were above 65 years old, and therefore, a calculated total of 150 participants could be recruited within 4 months.

### Interview

Two trained interviewers recruited and collected data on the day before surgery through face-to-face interviews in patients’ preferred medium of language (English, Malay, Chinese or Tamil) in the pre-operative admission ward. Demographic and surgical data were collected before proceeding to the specific questionnaires. Major surgery was defined according to the extent and complexity of the procedure, its pathophysiological consequences and consecutive clinical outcomes (Martin et al., 2020). For every interview, a specific question was asked if their surgery had been postponed because of the COVID-19 pandemic and limited elective operating services. The response was considered as postponed when either the patient replied ‘yes’ to the above or documented as such in the electronic medical records.

### Questionnaire instruments

For all participants, two validated questionnaires comprising Hospital Anxiety and Depression Scale (HADS) and 36-item Short Form Health Survey (SF-36) were used to assess their psychological status and overall quality of life respectively on the day before their surgery.

HADS is an established assessment composed of 14 items, seven questions for anxiety subscale (HADS Anxiety) and an equal number of questions for depression subscale (HADS Depression). It takes 2 to 5 min to complete referring to psychological symptoms experienced within the past week. Each question is scored using a 4-point Likert scale ranging from 0 to 3. The total score for each subscale will be summed up to give an outcome: a score of 0 to 7 as normal, 8 to 10 as borderline abnormal and 11 to 21 as abnormal (Snaith, 2003).

SF-36 is a well-researched, self-reported instrument originally developed to measure health-related quality of life at the individual level in clinical practice or health policy evaluations at the population level. There are 36 questions in this instrument covering eight health concepts: physical functioning, role limitations due to physical health problems, bodily pain, general health perceptions, vitality, social functioning, role limitations due to personal or emotional problems, and mental health (Lins & Carvalho, 2016).

For an overall completion of well-being assessment, a question was also asked regarding changes in appetite and responses were self-reported as increased, no change or decreased in this study.

### Data analysis

All statistical analyses were performed using IBM Statistical Package for the Social Sciences (SPSS) Statistics for Macintosh, Version 27.0. Demographic and survey responses were examined using frequency and percentages for categorical variables, mean and standard deviation (SD) or median and interquartile range (IQR) for continuous variables. The data were grouped into different categories to analyse for the difference between postponed and non-postponed surgical cases. We then performed subgroup analysis on the surgical cases that were postponed based on cancer and non-cancer-related cases. The Shapiro-Wilk test was used to examine the normality of data distribution. Normally distributed data were compared using the Student’s *t* test and non-normally distributed data were compared using the Mann–Whitney *U* test. All categorical data were analysed using chi-square test or Fisher’s exact test according to the test assumption. Univariable and multivariable logistic regression analyses were performed to assess the association between patients’ characteristics and anxiety and depression. Statistical significance was defined as *p* < 0.05.

## Results

### Postponed cases

One hundred and sixty patients were approached, but seven refused to participate, leading to a total of 153 geriatric patients with a mean (SD) age of 73 (±6) years old who consented and completed the interviews. Table 1 demonstrates the demographic data of this study population. A total of 36 out of 153 (23.5%) patients had their procedures deferred and returned for surgery during the first wave of COVID-19 pandemic.

**Table 1:** Demographic data of patients (*n* = 153)

Variables	Total (*n* = 153)	
	Number	%
Age group (years)		
65–74	103	67.3
75–84	42	27.5
≥ 85	8	5.2
Gender		
Male	77	50.3
Female	76	49.7
Ethnicity		
Malay	29	19.0
Chinese	97	63.4
Indian	24	15.7
Others	3	2.0
Diagnosis		
Non-cancer	98	64.1
Cancer	55	35.9
Nature of surgery		
Minor	85	55.6
Major	68	44.4
Surgical specialty		
Breast surgery	17	11.1
Colorectal surgery	8	5.2
Endocrine surgery	7	4.6
Hepatobiliary surgery	9	5.9
Upper gastrointestinal surgery	7	4.6
Cardiothoracic surgery	8	5.2
Gynaecology	10	6.5
Neurosurgery	6	3.9
Ophthalmology	34	22.2
Orthopaedic surgery	6	3.9
Otolaryngology	5	3.3
Plastic surgery	2	1.3
Urology	27	17.6
Vascular surgery	7	4.6
ASA physical status		
ASA I	2	1.3
ASA II	120	78.4
ASA III	31	20.3

*ASA* American Society of Anesthesiologists

Table 2 represents the analysis between the groups of patients who had their surgery postponed and the non-postponed cases. A significant difference was found between the two groups based on surgical specialty, diagnosis of cancer and nature of the operation (Table 2). Ophthalmology (*n* = 10, 27.8%) and urology (*n* = 14, 38.9%) had the most postponed cases. Similarly, minor surgeries (*n* = 28, 77.8%) were significantly postponed compared to major surgeries (*n* = 8, 22.2%) and non-cancer-related surgeries (*n* = 30, 83.3%) compared to those with cancer (*n* = 6, 16.7%). Although the number of recruited patients in breast surgery list was high (*n* = 17, 11.1%), only very few were previously postponed (*n* = 2, 5.6%).

**Table 2:** Patient and surgical-related characteristics based on non-postponed (*n* = 117) versus postponed cases (*n* = 36)

Variables	Non-postponed (*n* = 117)	Postponed (*n* = 36)	
	Number (%) or Median (IQR)	Number (%) or Median (IQR)	*p* value
Age group (years)			0.666
65–74	77 (65.8)	26 (72.2)	
75–84	33 (28.2)	9 (25.0)	
≥ 85	7 (6.0)	1 (2.8)	
Gender			0.139
Male	55 (47.0)	22 (61.1)	
Female	62 (53.0)	14 (38.9)	
Ethnicity			0.383
Malay	25 (21.4)	4 (11.1)	
Chinese	71 (60.7)	26 (72.2)	
Indian	18 (15.4)	6 (16.7)	
Others	3 (2.6)	0 (0.0)	
Diagnosis			0.006*
Non-cancer	68 (58.1)	30 (83.3)	
Cancer	49 (41.9)	6 (16.7)	
Nature of surgery			0.002*
Minor	57 (48.7)	28 (77.8)	
Major	60 (51.3)	8 (22.2)	
Surgical specialty			0.023*
Breast surgery	15 (12.8)	2 (5.6)	
Colorectal surgery	6 (5.1)	2 (5.6)	
Endocrine surgery	7 (6.0)	0 (0.0)	
Hepatobiliary surgery	7 (6.0)	2 (5.6)	
Upper gastrointestinal surgery	7 (6.0)	0 (0.0)	
Cardiothoracic surgery	7 (6.0)	1 (2.8)	
Gynaecology	10 (8.5)	0 (0.0)	
Neurosurgery	4 (3.4)	2 (5.6)	
Ophthalmology	24 (20.5)	10 (27.8)	
Orthopaedic surgery	5 (4.3)	1 (2.8)	
Otolaryngology	5 (4.3)	0 (0.0)	
Plastic surgery	1 (0.9)	1 (2.8)	
Urology	13 (11.1)	14 (38.9)	
Vascular surgery	6 (5.1)	1 (2.8)	
ASA physical status			0.720
ASA I	2 (1.7)	0 (0.0)	
ASA II	91 (77.8)	29 (80.6)	
ASA III	24 (20.5)	7 (19.4)	
HADS anxiety			0.640
Normal (0–7)	89 (76.1)	26 (72.2)	
Borderline abnormal (8–10) / Abnormal (11–21)	28 (23.9)	10 (27.8)	
Anxiety score	2.00 (0.00–7.00)	4.00 (0.00–8.00)	0.180
HADS depression			0.297
Normal (0–7)	105 (89.7)	30 (83.3)	
Borderline abnormal (8–10) / Abnormal (11–21)	12 (10.3)	6 (16.7)	
Depression score	0.00 (0.00–3.00)	1.00 (0.00–5.75)	0.424
SF-36 scale scores			
Physical functioning	75.00 (50.00–90.00)	70.00 (56.25–95.00)	0.882
Role limitations due to physical health	100.00 (0.00–100.00)	100.00 (81.25–100.00)	0.025*
Bodily pain	90.00 (68.75–100.00)	90.00 (67.50–100.00)	0.923
General health	65.00 (50.00–80.00)	60.00 (50.00–80.00)	0.666
Vitality	80.00 (65.00–90.00)	85.00 (60.00–95.00)	0.440
Social functioning	100.00 (100.00–100.00)	100.00 (100.00–100.00)	0.953
Role limitations due to emotional problems	100.00 (100.00–100.00)	100.00 (100.00–100.00)	0.404
Mental health	92.00 (80.00–96.00)	92.00 (80.00–99.00)	0.846
Appetite			0.153
Normal	100 (85.5)	34 (94.4)	
Decreased	17 (14.5)	2 (5.6)	

*ASA* American Society of Anesthesiologists, *HADS* Hospital Anxiety and Depression Scale, *SF-36* 36-item Short Form Health Survey, *IQR* interquartile range

Despite the lack of statistical significance, both the median HADS scores for anxiety and depression were higher for the group that had their surgeries postponed (2.00 [IQR 0.00–7.00] versus 4.00 [IQR 0.00–8.00] for anxiety, *p* = 0.180 and 0.00 [IQR 0.00–3.00] versus 1.00 [IQR 0.00–5.75] for depression, *p* = 0.424). The median scaled scores for SF-36 were not different except for role limitations secondary to physical health which had a tendency towards higher range of scores (*p* = 0.025). The complete results of specific HADS items for both anxiety and depression subscales will be presented in Supplementary File 1.

### Anxiety

Anxiety was present in both groups, those without their dates postponed (*n* = 28, 23.9%) and those with postponed dates (*n* = 10, 27.8%) although not statistically significant (*p* = 0.640). The factors associated with anxiety are demonstrated in Table 3. Patients who reported anxiety in this study also had significant risks for depression (odds ratio [OR] 24.35, 95% confidence interval [CI] 6.52–90.99, *p* < 0.001; adjusted OR 16.25, 95% CI 3.23–81.82, *p* = 0.001) and loss of appetite (OR 4.21, 95% CI 1.56–11.35, *p* = 0.005). Older patients ≥ 85 years old (OR 6.14, 95% CI 1.36–27.69, *p* = 0.018), female (OR 2.41, 95% CI 1.12–5.18, *p* = 0.024), cancer diagnosis (OR 2.19, 95% CI 1.04–4.64, *p* = 0.039) and scheduled for major surgery (OR 2.39, 95% CI 1.13–5.05, *p* = 0.023) were major predictive risks for anxiety. On the contrary, we found patients under ophthalmology (OR 0.14, 95% CI 0.04–0.51, *p* = 0.003; adjusted OR 0.08, 95% CI 0.01–0.54, *p* = 0.010) and urology (OR 0.11, 95% CI 0.02–0.53, *p* = 0.006; adjusted OR 0.09, 95% CI 0.01–0.69, *p* = 0.021) surgical divisions had lower risk for anxiety compared to other specialties.

**Table 3:** Univariable and multivariable models examining factors associated with anxiety

Covariates	Anxiety		Univariable		Multivariable	
	No Number (%)	Yes Number (%)	OR (95% CI)	*p* value	Adjusted OR (95% CI)	*p* value
Age group (years)						
65–74	81 (78.6)	22 (21.4)	1.00 (reference)		1.00 (reference)	
75–84	31 (73.8)	11 (26.2)	1.31 (0.57–3.00)	0.530	1.84 (0.58–5.82)	0.297
≥ 85	3 (37.5)	5 (62.5)	6.14 (1.36–27.69)	0.018*	1.94 (0.19–20.22)	0.579
Gender						
Male	64 (83.1)	13 (16.9)	1.00 (reference)		1.00 (reference)	
Female	51 (67.1)	25 (32.9)	2.41 (1.12–5.18)	0.024*	0.90 (0.30–2.69)	0.851
Ethnicity						
Malay	23 (79.3)	6 (20.7)	1.00 (reference)			
Chinese	73 (75.3)	24 (24.7)	1.26 (0.46–3.46)	0.653		
Indian	17 (70.8)	7 (29.2)	1.58 (0.45–5.55)	0.477		
Others	2 (66.7)	1 (33.3)	1.92 (0.15–24.87)	0.619		
Diagnosis						
Non-cancer	79 (80.6)	19 (19.4)	1.00 (reference)		1.00 (reference)	
Cancer	36 (65.5)	19 (34.5)	2.19 (1.04–4.64)	0.039*	0.44 (0.13–1.51)	0.194
Nature of surgery						
Minor	70 (82.4)	15 (17.6)	1.00 (reference)		1.00 (reference)	
Major	45 (66.2)	23 (33.8)	2.39 (1.13–5.05)	0.023*	1.12 (0.31–4.13)	0.862
Surgical speciality						
General surgery	28 (58.3)	20 (41.7)	1.00 (reference)		1.00 (reference)	
Cardiothoracic surgery	7 (87.5)	1 (12.5)	0.20 (0.02–1.76)	0.146	0.11 (0.01–1.52)	0.099
Gynaecology	8 (80.0)	2 (20.0)	0.35 (0.07–1.83)	0.213	0.32 (0.05–2.16)	0.240
Neurosurgery	4 (66.7)	2 (33.3)	0.70 (0.12–4.20)	0.696	0.43 (0.04–4.20)	0.467
Ophthalmology	31 (91.2)	3 (8.8)	0.14 (0.04–0.51)	0.003*	0.08 (0.01–0.54)	0.010*
Orthopaedic surgery	2 (33.3)	4 (66.7)	2.80 (0.47–16.80)	0.260	1.66 (0.13–21.63)	0.698
Otolaryngology	1 (20.0)	4 (80.0)	5.60 (0.58–53.95)	0.136	5.06 (0.39–65.58)	0.214
Plastic surgery	2 (100.0)	0 (0.0)	–		–	
Urology	25 (92.6)	2 (7.4)	0.11 (0.02–0.53)	0.006*	0.09 (0.01–0.69)	0.021*
Vascular surgery	7 (100.0)	0 (0.0)	–		–	
Postponement of surgery						
No	89 (76.1)	28 (23.9)	1.00 (reference)			
Yes	26 (72.2)	10 (27.8)	1.22 (0.53–2.84)	0.641		
Depression						
No	112 (83.0)	23 (17.0)	1.00 (reference)		1.00 (reference)	
Yes	3 (16.7)	15 (83.3)	24.35 (6.52–90.99)	< 0.001*	16.25 (3.23–81.82)	0.001*
Appetite						
Normal	106 (79.1)	28 (20.9)	1.00 (reference)		1.00 (reference)	
Decreased	9 (47.4)	10 (52.6)	4.21 (1.56–11.35)	0.005*	2.41 (0.57–10.12)	0.231

*OR* odds ratio, *CI* confidence interval

### Depression

A total of twelve patients (10.3%) who underwent surgery without any delay and six (16.7%) who were rescheduled reported depression although no statistical significance was shown (*p* = 0.297). However, significant association was found between anxiety and depression in the univariate and multivariate logistic regression analyses for depression in Table 4 (OR 24.35, 95% CI 6.52–90.99, *p* < 0.001; adjusted OR 16.62, 95% CI 4.12–66.99, *p* < 0.001). Patients who reported depression were also likely to have loss of appetite (OR 6.52, 95% CI 2.13–19.95, *p* = 0.001). The other significant risk factors for depression were age ≥ 85 years (OR 10.44, 95% CI 2.22–48.99, *p* = 0.003) and female (OR 6.07, 95% CI 1.68–21.93, *p* = 0.006; adjusted OR 4.60, 95% CI 1.02–20.68, *p* = 0.047).

**Table 4:** Univariable and multivariable models examining factors associated with depression

Covariates	Depression		Univariable		Multivariable	
	No Number (%)	Yes Number (%)	OR (95% CI)	*p* value	Adjusted OR (95% CI)	*p* value
Age group (years)						
65–74	94 (91.3)	9 (8.7)	1.00 (reference)		1.00 (reference)	
75–84	37 (88.1)	5 (11.9)	1.41 (0.44–4.49)	0.560	1.21 (0.28–5.32)	0.801
≥ 85	4 (50.0)	4 (50.0)	10.44 (2.22–48.99)	0.003*	4.23 (0.45–39.86)	0.207
Gender						
Male	74 (96.1)	3 (3.9)	1.00 (reference)		1.00 (reference)	
Female	61 (80.3)	15 (19.7)	6.07 (1.68–21.93)	0.006*	4.60 (1.02–20.68)	0.047*
Ethnicity						
Malay	26 (89.7)	3 (10.3)	1.00 (reference)			
Chinese	87 (89.7)	10 (10.3)	1.00 (0.26–3.89)	0.996		
Indian	19 (79.2)	5 (20.8)	2.28 (0.49–10.73)	0.297		
Others	3 (100.0)	0 (0.0)	–			
Diagnosis						
Non-cancer	90 (91.8)	8 (8.2)	1.00 (reference)			
Cancer	8 (81.8)	10 (18.2)	2.50 (0.92–6.77)	0.071		
Nature of surgery						
Minor	79 (92.9)	6 (7.1)	1.00 (reference)			
Major	56 (82.4)	12 (17.6)	2.82 (1.00–7.97)	0.050		
Surgical specialty						
General surgery	40 (83.3)	8 (16.7)	1.00 (reference)			
Cardiothoracic surgery	7 (87.5)	1 (12.5)	0.72 (0.08–6.63)	0.767		
Gynaecology	9 (90.0)	1 (10.0)	0.56 (0.06–5.02)	0.601		
Neurosurgery	5 (83.3)	1 (16.7)	1.00 (0.10–9.75)	1.000		
Ophthalmology	32 (94.1)	2 (5.9)	0.31 (0.06–1.58)	0.159		
Orthopaedic surgery	4 (66.7)	2 (22.2)	2.50 (0.39–16.05)	0.334		
Otolaryngology	3 (60.0)	2 (40.0)	3.33 (0.48–23.28)	0.225		
Plastic surgery	2 (100.0)	0 (0.0)	–			
Urology	26 (96.3)	1 (3.7)	0.19 (0.02–1.63)	0.130		
Vascular surgery	7 (100.0)	0 (0.0)	–			
Postponement of surgery						
No	105 (89.7)	12 (10.3)	1.00 (reference)			
Yes	30 (83.3)	6 (16.7)	1.75 (0.61–5.05)	0.301		
Anxiety						
No	112 (97.4)	3 (2.6)	1.00 (reference)		1.00 (reference)	
Yes	23 (60.5)	15 (39.5)	24.35 (6.52–90.99)	< 0.001*	16.62 (4.12–66.99)	< 0.001*
Appetite						
Normal	123 (91.8)	11 (8.2)	1.00 (reference)		1.00 (reference)	
Decreased	12 (63.2)	7 (36.8)	6.52 (2.13–19.95)	0.001*	3.88 (0.93–16.29)	0.064

*OR* odds ratio, *CI* confidence interval

### Cancer

From the 36 postponed cases, 6 were cancer-related, as shown in Table 5. They comprised surgical specialties in breast (*n* = 2), colorectal (*n* = 1), plastic (*n* = 1) and urology (*n* = 2). Besides the significant difference in general health and vitality, the fear of disease progression after the postponement was significantly more frequent in cancer patients (*p* = 0.035). Two patients developed bone metastases with repeat bioimaging and another had evidence of increase in the size of the primary tumour over the 3 months after their first scheduled date.

**Table 5:** Postponed cases by diagnosis (*n* = 36)

Variables	Non-cancer (*n* = 30)	Cancer (*n* = 6)	
	Number (%) or Median (IQR)	Number (%) or Median (IQR)	*p* value
Age group (years)			0.806
65–74	22 (73.3)	4 (66.7)	
75–84	7 (23.3)	2 (33.3)	
≥ 85	1 (3.3)	0 (0.0)	
Gender			0.541
Male	19 (63.3)	3 (50.0)	
Female	11 (36.7)	3 (50.0)	
Ethnicity			0.362
Malay	4 (13.3)	0 (0.0)	
Chinese	22 (73.3)	4 (66.7)	
Indian	4 (13.3)	2 (33.3)	
Nature of surgery			0.073
Minor	25 (83.3)	3 (50.0)	
Major	5 (16.7)	3 (50.0)	
Surgical specialty			0.018*
Breast surgery	0 (0.0)	2 (33.3)	
Colorectal surgery	1 (3.3)	1 (16.7)	
Endocrine surgery	0 (0.0)	0 (0.0)	
Hepatobiliary surgery	2 (6.7)	0 (0.0)	
Upper gastrointestinal surgery	0 (0.0)	0 (0.0)	
Cardiothoracic surgery	1 (3.3)	0 (0.0)	
Gynaecology	0 (0.0)	0 (0.0)	
Neurosurgery	2 (6.7)	0 (0.0)	
Ophthalmology	10 (33.3)	0 (0.0)	
Orthopaedic surgery	1 (3.3)	0 (0.0)	
Otolaryngology	0 (0.0)	0 (0.0)	
Plastic surgery	0 (0.)	1 (16.7)	
Urology	12 (40.0)	2 (33.3)	
Vascular surgery	1 (3.3)	0 (0.0)	
ASA physical status			0.187
ASA I	0 (0.0)	0 (0.0)	
ASA II	23 (76.7)	6 (100.0)	
ASA III	7 (23.3)	0 (0.0)	
HADS anxiety			0.183
Normal (0–7)	23 (76.7)	3 (50.0)	
Borderline abnormal (8–10) / Abnormal (11–21)	7 (23.3)	3 (50.0)	
Anxiety score	4.00 (0.00–7.25)	7.00 (0.75–10.00)	0.467
HADS depression			1.000
Normal (0–7)	25 (83.3)	5 (83.3)	
Borderline abnormal (8–10) / Abnormal (11–21)	5 (16.7)	1 (16.7)	
Depression score	0.50 (0.00–2.75)	4.00 (0.75–7.50)	0.186
SF-36 scale scores			
Physical functioning	70.00 (58.75–96.25)	70.00 (38.75–91.25)	0.576
Role limitations due to physical health	100.00 (100.00–100.00)	75.00 (0.00–100.00)	0.201
Bodily pain	90.00 (67.50–100.00)	83.75 (52.50–100.00)	0.852
General health	70.00 (55.00–86.25)	50.00 (41.25–56.25)	0.016*
Vitality	85.00 (73.75–95.00)	52.50 (45.00–85.00)	0.018*
Social functioning	100.00 (100.00–100.00)	93.75 (56.25–100.00)	0.233
Role limitations due to emotional problems	100.00 (100.00–100.00)	100.00 (0.00––100.00)	0.394
Mental health	92.00 (80.00–97.00)	80.00 (62.00–100.00)	0.493
Appetite			0.193
Normal	29 (96.7)	5 (83.3)	
Decreased	1 (3.3)	1 (16.7)	
Fear of disease progression due to surgery postponement			0.035*
No	23 (76.7)	2 (33.3)	
Yes	7 (23.3)	4 (66.7)	

*ASA* American Society of Anesthesiologists, *HADS* Hospital Anxiety and Depression Scale, *SF-36* 36-item Short Form Health Survey, *IQR* interquartile range

## Discussion

During the first pandemic declaration in March 2020, rescheduling elective surgeries began by allowing only limited semi-emergency lists, emergency and obstetric cases in this institution to proceed as a COVID-19 hybrid medical centre. The operating theatre (OT) was reduced to less than 30% to channel workforce, resources and hospital beds to manage the increasing number of COVID-19 patients in this region. After 8 weeks, the OT capacity was gradually increased to its routine with reinforced protocols for patient and healthcare safety.

This study demonstrated that 23.5% of elective surgeries were postponed cases from March 2020. These postponed elective cases were minor and non-cancer-related surgeries as recommended by international guidelines to triage procedures in the OT lists. Following that, the American College of Surgeons published guiding principles to lead institutions in making decisions based on surgical prioritization during the pandemic phase while, at the same time, conserved manpower and resources (American College of Surgeons, 2020a). Retrospectively, we experienced Acute Phase 1 during the first OT closure and relied on identifying and performing surgeries that were needed within 3 months or otherwise survivorship would have been compromised. All emergency and semi-urgent cases such as cancer-related surgeries were given priorities as recommended (Federation of Surgical Specialty Associations, 2021; European Society for Medical Oncology, 2020; American College of Surgeons, 2020b).

The highest number of rescheduled cases was notably from surgical specialties such as ophthalmology and urology since the profile of their patients was usually older, which was also the main  inclusion criteria in this study. Furthermore, lens and cataract removal had always been shown to be the most common procedure for adults aged above 65 years old (Deiner et al., 2014). And because this procedure is clearly defined as minor, most of them would have been postponed. Similarly, urology tends to have older patients and minor procedures such as transurethral resection of prostate or removal of urinary obstruction, another common surgery for geriatric patients. Hence, the same clinical decision was made for patients under this team during the OT semi-closure.

Although countries around the world were compelled to postpone or cancel their surgical lists during the same period, reports related to the medical impact or patients’ outcome remained scarce and variable. Surgical departments in two German university hospitals concluded that no disproportionate impact on patient outcomes occurred in their institutions while averting nosocomial transmission of COVID-19 with well-organized and early suspension of elective surgery (Metelmann & Busemann, 2020). Their elective surgery was completely suspended from March to April 2020 with an average time of postponement differing from 47 to 124 days. However, it would not be appropriate to make a fair comparison of their analysis to the current study results since both were relatively small centres of less than a hundred beds, had low COVID-19 infection rates and maintained reliable capacities for ventilation of ill patients and intensive care unit (ICU) beds throughout the epidemic (Deiner et al., 2014). In Italy, at the peak of the epidemic curve when 91.3% of the ICU beds were used for COVID-19 patients, elective surgery reduced by 75.0% while urgent or emergency surgery then decreased by 30.0% (Di Marzo et al., 2020). The study reported short-term impact of surgeries performed, looking into significantly higher median operative time, stay in OT and rate of patients with Clavien-Dindo grade 3b postoperative complications requiring a second procedure within 7 days but not the effect on rescheduled cases after that cancellation period (Dindo et al., 2004).

Based on the interview findings, we found that anxiety and depression were not uncommon especially in the elderly above 85 years old and females which were similar to previous studies (Mirani et al., 2019; Löbner et al., 2012). Both forms of psychological distress also resulted in significant loss of appetite. Despite the lack of statistical significance, the HADS scores for both anxiety and depression were higher in the group with postponement from their original dates. Although prevalent, peri-operative anxiety and depression are often under-reported and overlooked in vulnerable individuals displaying subtle geriatric-specific syndromes (Kim et al., 2015; Byers et al., 2010). Furthermore, when anxiety was present, the risk for depression would follow with a significant rise and vice versa as shown in our study.

In the past, these psychological disorders were mostly due to inadequate adjustment to unfamiliar hospital environment, insufficient privacy, exposure to strange instruments, financial concerns and disease stress (Mirani et al., 2019). But for the months preceding this study, a new concern for older surgical patients developed and profoundly impacted their well-being (Arjomandi Rad & Vardanyan, 2020). Strict lockdowns, social isolation and lack of support in addition to fear of contracting the COVID-19 infection from both community and within hospitals while seeking treatment compounded to the existing reasons for deteriorating mental health (Arjomandi Rad & Vardanyan, 2020). Postponement of their surgeries caused further harm ‘downstream’ in the consequence of waiting for a new date and managing the disease at home (Brown et al., 2021). Even so, with a lengthy waitlist despite resuming elective surgeries, patients continue to experience clinical depression that could lead to disruption of their daily activities (The Lancet R, 2021). Moreover, anxiety and depression peri-operatively are psychological factors associated with surgical recovery, length of hospitalization, readmission rates and mortality of the older patient (Abraham et al., 2020; Ghoneim & O'Hara, 2016; Singleton & Poutawera, 2017). Even though it is important to be cognizant of peri-operative psychological distress, this study however, did not demonstrate the impact of postponement on HADS scores in a statistically significant manner.

Nevertheless, recommendations to acknowledge the psychological symptoms early is still recommended as the first step, followed by simple screening tools to confirm and discuss coping strategies with patients and family (Arjomandi Rad & Vardanyan, 2020). Another solution suggested by experts is to prioritize novel approaches in telemedicine (Hildrew, 2020). This will avoid person-to-person contact and yet preserve the continuum of care for some form of psychological support intervention in a prehabilitation bundle for their health management by general or specialized practitioners (Arjomandi Rad & Vardanyan, 2020; Xiao et al., 2017; Tsimopoulou et al., 2015).

A particular subgroup of patients during this period of COVID-19 who had imposed critical challenges to medical teams were cancer patients (Aminian et al., 2020). The surgical management in cancer patients may be more complicated, especially in those who were clinically frail and ill from the capacitating disease itself or other co-morbidities. Thereby, exposing these patients to peri-operative risks will necessitate intensive postoperative care, longer hospitalizations and concurrently raise their susceptibility to both common infections and COVID-19 transmission (Di Saverio et al., 2020; Sharma et al., 2020). From the results, most postponed cases were non-cancer related. Only a few were cancer-related surgeries from breast, colorectal, plastic and urology specialties that were likely to be long surgeries, requiring the already exhausted critical care support and resources such as blood supply.

In some cancers, surgical deferment can be considered, particularly if alternative treatments are available. For example, in breast cancer, adopting neoadjuvant chemotherapy and hormonal therapy has been proposed (Sheng et al., 2020). On the other hand, the timing may have a significant impact on prognosis and subsequent quality of life in cancer patients (Samson et al., 2015; Grotenhuis et al., 2010; van Harten et al., 2015). The decision to proceed or postpone can be challenging and should be done with care from a multi-disciplinary approach by considering all the aspects of each case including the type and stage of cancer, the age, the physical status, the psychological issues and other treatments (Soltany et al., 2020). There was definitely fear among cancer cases that had to be postponed for the spread of their disease and evidence that the disease has progressed unfavourably. Nevertheless, the knowledge regarding cancer surgery and oncology management is rapidly evolving. It is on the onus of the primary teams, as a whole, to decide and adopt the principle of a global approach for specific cancers versus a case-by-case approach as recommended by the Society of Surgical Oncology amidst the restricted hospital access and resources in the present state (Bartlett et al., 2020).

This study has several limitations. First of all, data of the exact number of postponed surgeries were not collected, and the cases captured here may represent only a fraction of the rescheduled cases. Patients who had decided to undergo their procedures in other institutions or those who had succumbed to death while on the waiting list were not captured in this study. Among oncological cases, some were also referred to nearby centres for surgery to mitigate the long wait or risks of cancellation which might have caused a certain degree of selection bias. Secondly, the low number of postponed cases included in this sample especially for cancer-related diagnosis limits conclusions and generalizability of the findings. Patient sampling was also limited to the period of 3 months when elective surgery restarted, done in a single centre and did not include emergency cases. Hence, postponed patients who ended up requiring urgent procedures because of complications in their primary diagnosis could have been missed. Finally, we did not explore specific fears in patients towards COVID-19 as a cause of their anxiety and depression.

However, this study focused mainly on the clinical and psychological impact of older patients who underwent elective procedures when OT was resumed following the lockdown. Across the globe, many institutions would have likely experienced the same dilemma when millions of operations were cancelled or postponed to enhance capacity to manage the peak of COVID-19 cases in 2019-2020 (COVIDSurg Collaborative, 2020). In the aftermath of managing the pandemic, a massive backlog of surgical cases became a repercussion to many older patients, increasing their anxiety, depression and fear. As a result, their elective surgeries became ‘too long to wait’ for (The Lancet R, 2021). As the healthcare system moved towards handling wave after wave of the pandemic, drastic measures for surgical prioritization and redirection of medical attention continued to dilute and prevent the escalation of the much-needed care in non-COVID-19 patients.

## Conclusion

A number of cases who were postponed and rescheduled after the first wave of COVID-19 had both anxiety and depression, especially in cancer patients. Results of this study may represent only a tip of the iceberg as each cycle of lockdown and OT cancellation during the last 2 years increased the risks of developing these symptoms in waiting patients. When cases are postponed in elective surgeries, factors to consider should include older patients, their disease progression and the psychological impact caused by the delay. In peri-operative medicine, an appropriate and holistic management for postponed geriatric surgical cases with the incorporation of telemedicine will be the new direction in our anaesthetic practice.

## Supplementary Information

**Additional file 1.** Results of specific HADS items for both anxiety and depression subscales (*n* = 153). Values are number (proportion).

## Data Availability

The datasets used and analysed during this study are available from the corresponding author on reasonable request.

## Abbreviations

CI: Confidence interval
COVID-19: Coronavirus disease 2019
GA: General anaesthesia
HADS: Hospital Anxiety and Depression Scale
ICU: Intensive care unit
IQR: Interquartile range
MAC: Monitored anaesthetic care
MMSE: Mini-Mental State Examination
OR: Odds ratio
OT: Operating theatre
RA: Regional anaesthesia
SARS-CoV-2: Severe acute respiratory syndrome coronavirus 2
SD: Standard deviation
SF-36: 36-item Short Form Health Survey
SPSS: Statistical Package for the Social Sciences
STROBE: Strengthening the Reporting of Observational Studies in Epidemiology
