# Supplementary material for: Psychological distress among elderly surgical patients who had their surgery postponed during the COVID-19 pandemic
Source: Perioper Med (Lond). 2022 Mar 17;11:10. doi: 10.1186/s13741-022-00242-7 (PMC8926453; doi:10.1186/s13741-022-00242-7)
Supplement: Supplementary file 1 — Additional file 1. Results of specific HADS items for both anxiety and depression subscales (n = 153). Values are number (proportion). [file 13741_2022_242_MOESM1_ESM.docx]

**Supplementary File 1** Results of specific HADS items for both anxiety and depression subscales (n = 153). Values are number (proportion).

| **HADS Item** | **Text of Items** | **Not postponed**  **(n = 117)** | **Postponed**  **(n = 36)** |
| --- | --- | --- | --- |
| **Anxiety subscale** | | | |
| (1) | I feel tense or “wound up”  Most of the time  A lot of the time  Time to time, occasionally  Not at all | 4 (3.4)  9 (7.7)  41 (35.0)  63 (53.8) | 3 (8.3)  4 (11.1)  15 (41.7)  14 (38.9) |
| (3) | I get a sort of frightened feeling as if something awful is about to happen  Very definitely and quite badly  Yes, but not too badly  A little, but it doesn’t worry me  Not at all | 5 (4.3)  21 (17.9)  26 (22.2)  65 (55.6) | 2 (5.6)  7 (19.4)  11 (30.6)  16 (44.4) |
| (5) | Worrying thoughts go through my mind  A great deal of the time  A lot of the time  From time to time but not too often  Only occasionally | 4 (3.4)  12 (10.3)  26 (22.2)  75 (64.1) | 3 (8.3)  4 (11.1)  8 (22.2)  21 (58.3) |
| (7) | I can sit at ease and feel relaxed  Definitely  Usually  Not often  Not at all | 77 (65.8)  22 (18.8)  12 (10.3)  6 (5.1) | 22 (61.1)  5 (13.9)  7 (19.4)  2 (5.6) |
| (9) | I get a sort of frightened feeling like ‘‘butterflies’’ in the stomach  Not at all  Occasionally  Quite often  Very often | 66 (56.4)  39 (33.3)  6 (5.1)  6 (5.1) | 14 (38.9)  16 (44.4)  5 (13.9)  1 (2.8) |
| (11) | I feel restless as if I have to be on the move  Very much indeed  Quite a lot  Not very much  Not at all | 1 (0.9)  11 (9.4)  31 (26.5)  74 (63.2) | 1 (2.8)  4 (11.1)  8 (22.2)  23 (63.9) |
| (13) | I get sudden feelings of panic  Very often indeed  Quite often  Not very often  Not at all | 5 (4.3)  13 (11.1)  33 (28.2)  66 (56.4) | 1 (2.8)  3 (8.3)  13 (36.1)  19 (52.8) |
|  |  |  |  |
| **Depression subscale** | | | |
| (2) | I still enjoy the things I used to enjoy  Definitely as much  Not quite so much  Only a little  Hardly at all | 84 (71.8)  19 (16.2)  12 (10.3)  2 (1.7) | 26 (72.2)  7 (19.4)  2 (5.6)  1 (2.8) |
| (4) | I can laugh and see the funny side of things  As much as I always could  Not quite so much now  Definitely not so much now  Not at all | 97 (82.9)  17 (14.5)  3 (2.6)  0 (0.0) | 29 (80.6)  1 (2.8)  3 (8.3)  3 (8.3) |
| (6) | I feel cheerful  Not at all  Not often  Sometimes  Most of the time | 1 (0.9)  9 (7.7)  18 (15.4)  89 (76.1) | 0 (0.0)  3 (8.3)  7 (19.4)  26 (72.2) |
| (8) | I feel as if I am slowed down  Nearly all the time  Very often  Sometimes  Not at all | 4 (3.4)  4 (3.4)  37 (31.6)  72 (61.5) | 0 (0.0)  4 (11.1)  10 (27.8)  22 (61.1) |
| (10) | I have lost interest in my appearance  Definitely  I don’t take so much care as I should  I may not take quite as much care  I take just as much care as ever | 0 (0.0)  2 (1.7)  13 (11.1)  102 (87.2) | 1 (2.8)  1 (2.8)  3 (8.3)  31 (86.1) |
| (12) | I look forward with enjoyment to things  As much as I ever did  Rather less than I used to  Definitely less than I used to  Hardly at all | 93 (79.5)  18 (15.4)  4 (3.4)  2 (1.7) | 28 (77.8)  4 (11.1)  2 (5.6)  2 (5.6) |
| (14) | I can enjoy a good book or radio or TV programme  Often  Sometimes  Not often  Very seldom | 92 (78.6)  16 (13.7)  5 (4.3)  4 (3.4) | 25 (69.4)  9 (25.0)  2 (5.6)  0 (0.0) |

HADS, Hospital Anxiety and Depression Scale
